# Hair Follicle Dermal Cells Support Expansion of Murine and Human Embryonic and Induced Pluripotent Stem Cells and Promote Haematopoiesis in Mouse Cultures

**DOI:** 10.1155/2018/8631432

**Published:** 2018-08-02

**Authors:** Jun Liu, Claire A. Higgins, Jenna C. Whitehouse, Susan J. Harris, Heather Crawford, Angela M. Christiano, Majlinda Lako, Nicholas Hole, Colin A. B. Jahoda

**Affiliations:** ^1^Department of Biosciences, Durham University, Durham DH1 3LE, UK; ^2^Department of Dermatology, Columbia University, 1150 St Nicholas Avenue, New York, NY 10032, USA; ^3^Institute of Human Genetics, International Centre for Life, Newcastle University, Central Parkway, Newcastle upon Tyne NE1 3BZ, UK

## Abstract

In the hair follicle, the dermal papilla (DP) and dermal sheath (DS) support and maintain proliferation and differentiation of the epithelial stem cells that produce the hair fibre. In view of their regulatory properties, in this study, we investigated the interaction between hair follicle dermal cells (DP and DS) and embryonic stem cells (ESCs); induced pluripotent stem cells (iPSCs); and haematopoietic stem cells. We found that coculture of follicular dermal cells with ESCs or iPSCs supported their prolonged maintenance in an apparently undifferentiated state as established by differentiation assays, immunocytochemistry, and RT-PCR for markers of undifferentiated ESCs. We further showed that cytokines that are involved in ESC support are also expressed by cultured follicle dermal cells, providing a possible explanation for maintenance of ES cell stemness in cocultures. The same cytokines were expressed within follicles *in situ* in a pattern more consistent with a role in follicle growth activities than stem cell maintenance. Finally, we show that cultured mouse follicle dermal cells provide good stromal support for haematopoiesis in an established coculture model. Human follicular dermal cells represent an accessible and readily propagated source of feeder cells for pluripotent and haematopoietic cells and have potential for use in clinical applications.

## 1. Introduction

Adult hair follicle dermal cell populations have extensive regenerative, inductive, and supportive capabilities, both within adult and developing hair follicles [1, 2] and in combination with other cell types including cornea and amnion [3, 4]. Experimentally, subpopulations of adult hair follicle dermal cells have demonstrated extensive stem cell capabilities, and multipotency, including generation of bone, fat, and muscle *in vitro* [5–7]. Additionally, dermal cells can differentiate down a haematopoietic lineage both *in vivo* and *in vitro*, [8, 9] and have characteristics similar to embryonic neural crest stem cells [10]. In this respect, they behave similarly to stem cell populations isolated from adult bone marrow, a common source of adult stem cells [11]. It is interesting to note that cells isolated from adult bone marrow also have supportive capabilities, particularly in the support of hematopoietic stem cells and embryonic stem cells (ESCs) *in vitro* [12–14]. Bone marrow cells support epidermal keratinocytes in *in vitro* skin reconstitution assays [15] and during cutaneous wound healing [16], demonstrating significant similarities with hair follicle dermal cells [17, 18].

ESCs, derived from the inner cell mass of mammalian blastocysts [19–21], retain their developmental potential after prolonged culture to differentiate down all three germ layer lineages *in vivo* and *in vitro*. Induced pluripotent stem cells (iPSCs), which are almost comparable to ESCs, are generated by reprogramming somatic cells, a process initially achieved using virus-mediated gene transduction of a few key factors [22, 23]. While mouse ESCs (mESCs) can be continuously cultured without feeder cells on gelatin-coated plates with the addition of leukemia inhibitory factor (LIF) [24], human ESCs (hESCs) or iPSCs will differentiate in culture in the presence of this cytokine. Propagation of undifferentiated human ESCs or iPSCs is commonly carried out by coculture with a mouse embryonic fibroblast (MEF) feeder layer. To improve the potential clinical utility of human pluripotent cells, considerable progress has been made in establishing defined feeder-free culture systems for hESCs or iPSCs [25–27], with methods that include growing the cells on specific substrates [28, 29], in suspension cultures [30] or in defined serum-free medium [31]. Another approach has been to replace MEFs with human-derived cells. For example, bone marrow stromal cells (BMSC) [13], neonatal skin fibroblasts, stromal cells [32–34], amniotic mesenchymal cells [35], or human foetal cell lines [36] have all been employed as feeder cells. However, the use of human feeder layers requires that the feeder cell type is easily accessible, readily propagated, and efficient at maintaining and amplifying undifferentiated hESCs suitable for clinical use.

Interactions between adjacent cells of different types are a major mechanism of organogenesis in developmental biology. In view of the inductive properties of hair follicle dermal cells [1, 2], we initially set out to investigate their effects on mESCs by coculture *in vitro*, anticipating that the dermal cells would exert some directive influence on mESC differentiation. However, we found that follicular dermal cells appeared to be effective in maintaining the mESCs in an undifferentiated state. This was confirmed by inducing differentiation of the mESCs along multiple lineages after prolonged coculture and by investigating the expression of markers characteristic of undifferentiated mESCs by RT-PCR and immunofluorescence. We subsequently investigated the mechanism by which dermal cultures may be able to support mESCs by examining the expression of members of the IL-6 family of cytokines, known to be crucial for maintaining murine embryonic stem cell pluripotency *in vitro* via the gp130 receptor and the JAK/STAT pathway. Parallel investigations were also performed on follicles, based on the hypothesis that follicle epithelial stem cells might be maintained in an undifferentiated state by ES cell-type mechanisms. This was not supported by the observations, but the prevalence of IL-6 family cytokines and the gp130 receptor in follicles did point to a functional role of gp130/JAK/STAT signalling in hair follicle activities. When the ability of human hair follicle dermal cells to maintain hESCs and hiPSCs in an undifferentiated state was assessed, it was confirmed that like their rodent cell counterparts, the follicle dermal cells were superior to skin fibroblasts in their ability to maintain and support hESC and iPSC cultures. Finally, given the apparent similarities between bone marrow stromal cells and hair follicle dermis/mesenchyme [17], we performed coculture experiments to investigate the ability of hair follicle dermal cells to support haematopoietic activity. Here again, the follicle cells were the equal if not better than bone marrow-derived stromal cells under the experimental conditions employed.

These observations have implications for the regulation of both dermal and epithelial stem cells in the hair follicle, as well as confirming that hair follicle dermal cells have the potential to be a useful source of feeder cells for the support and amplification of a range of stem cell types.

## 2. Materials and Methods

### 2.1. Hair Follicle DP and DS Cell Isolation and Culture

DP and DS were microdissected from the vibrissa follicles of adult PVG rats or BalbC or Zin40 mice as previously described [37]. Animal tissues were obtained from animals housed in accordance with the institutional guidelines at the University of Durham. Human DP and DS were microdissected from skin biopsies as previously described [2], with skin biopsies obtained as anonymised discarded tissue in accordance with Helsinki guidelines. Skin dermal fibroblast (SF) cultures were established as explants from finely minced rodent footpad or human interfollicular scalp skin. A spontaneously transformed rat dermal papilla cell line, RDP-B [38], was also used as a control line. Once established, cells were maintained in MEM (Sigma) supplemented with 10% FBS (Gibco) and antibiotics (Sigma) (dermal cell medium) at 37°C, 5% CO_2_, with passaging every 2–4 weeks.

### 2.2. Mouse ESC Culture

Mouse CGR8 ESCs were routinely cultured on mitomycin C-inactivated MEF feeder layers in Glasgow MEM supplemented with 10% FBS, 100 *μ*M *β*-mercaptoethanol (Gibco), 2 mM L-glutamine, 1% nonessential amino acids, 0.25% NaHCO3, 1 mM pyruvate (Sigma), and 1000 U/ml LIF (Chemicon) (mESC medium). Cells were grown on 0.1% gelatin (Sigma) coated 6-well plates and split at ratios from 1 : 3 and 1 : 6 to 1 : 8 prior to becoming confluent. Two CGR8 cell lines transfected with GFP-expressing vectors were used in various assays; one designated CGR8-GFP, expressed GFP from a CAGG promoter which escaped silencing in mESCs, and was used to track both differentiated and undifferentiated mESCs in cocultures, while the second designated CGR8 Rex1-EGFP, expressed GFP under the control of the Rex-1 promoter, and was used to localize undifferentiated mESCs in cocultures. mESCs were stably transfected with the Rex 1-EGFP vector (a kind gift from Dr. N. Benvenisty) [39], using TransFast (Promega) in accordance with the manufacturer's protocol. CGR8 Rex1-EGFP cells were routinely maintained on MEF feeder layers in mESC medium with periodic reselection by addition of 400 *μ*g/ml G418 to the culture medium.

### 2.3. 2D Coculture of mESCs with Rodent Dermal Cells

The CGR8 Rex1-EGFP and CGR8-GFP cells were used for coculture experiments. Initially, rodent DP, DS, or SF cells were plated at 4 × 10^5^ cells per well of 6-well culture plates (the same cell density as MEFs for feeder layers). Subconfluent mESCs were then seeded over the feeder layer in each well. The rodent cocultures were passaged every two days with a splitting ratio of 1 : 6. A second set of experiments was performed in which rodent DP or DS monolayers were established in 35 mm culture dishes, and 500, 3000, 5000, or 10,000 Rex 1-EGFP mESCs were added to each dish when the dermal layer reached 80% confluence. These cocultures were then maintained for up to 4 weeks without splitting. A third set of cocultures was also performed but dermal cells were physically separated from mESCs by seeding them on porous membrane inserts (0.45 *μ*m pore size, Falcon), placed over 6-well plates containing mESCs.

All cocultures were routinely maintained in dermal cell medium, without the addition of LIF, but some samples were also grown in ESC differentiation medium (mESC medium without LIF). At least 3 sets of dermal/ES cell cocultures were established, and in the case of the hair follicle dermal cells, experiments were repeated at least 6 times. Dermal cells used were between passage 2 and passage 7 with over 30% of them at passage 1 to passage 3.

### 2.4. Culture of mESCs with Dermal Cell-Conditioned Medium

To obtain conditioned medium, rodent dermal cells or dermal/ES cell cocultures were incubated with dermal cell medium for 48 hours. After this, time medium was collected and centrifuged (30 min, 3300 rpm) to remove cell debris and the supernatant were stored at −20°C for future use. mESCs were then cultured for 10 days in conditioned medium (CM) diluted 1 : 1 with ESC differentiation medium. The morphology of these cells was compared to mESCs cultured for the same period in mESC medium with or without LIF.

### 2.5. 3D Coculture of Rodent Cells

CGR8 mESCs and PVG rat DP or DS cells were mixed in three different ratios of mESC : dermal cells (1 : 1, 1 : 3, and 1 : 10). 3D cultures were generated using a hanging drop method; 10 *μ*l drops of cell suspension in ESC differentiation medium containing approximately 400 mixed cells were placed on the lids of bacteriological Petri (nontissue culture-treated) dishes filled with phosphate buffered saline (PBS). Several sets of cocultures utilized follicular dermal cells stained with DiI and CGR8-GFP mESCs so that the mESC and follicular dermal cells could be distinguished in mixed cultures. After cultivation for 3 days, the aggregates were transferred into bacteriological Petri dishes and maintained in suspension in ESC differentiation medium. Differentiation was investigated by microscopy, immunofluorescence, and RT-PCR of the embryoid-like bodies after a further 9 days of culture.

### 2.6. Differentiation of mESC Cultures

CGR8-GFP mESCs were isolated from cocultures with DP or DS cells. The medium was removed from the cocultures and the cells were washed twice with PBS. A 30 *μ*l volume of TVP solution (trypsin, versene, and chick plasma; Sigma) was taken up in a 200 *μ*l automatic pipette tip and repeatedly expelled and aspirated over a small area of the coculture until gaps could be seen in the cell layer. The contents of the tip were then expelled into a gelatin-coated 35 mm culture dish, and the cells were briefly expanded in mESC medium prior to being plated at 0.54 × 10^6^ cells per 90 mm bacteriological Petri dish to generate EBs. After a short suspension period, EBs could be observed; medium was changed every 2 days by centrifuging EBs for 3 minutes at 800 rpm and resuspending in fresh ESC differentiation medium. EBs were then exposed to different conditions to promote differentiation.

#### 2.6.1. Neuronal Differentiation Assay

4 days after EB establishment, all-trans-retinoic acid (Sigma), diluted in ESC differentiation medium, was added to the suspension cultures (at a final concentration of 10^−7^ M). This was repeated on day 6. By day 8, approximately 50 EBs were transferred to gelatin-coated 60 mm dishes and incubated with ESC differentiation medium without *β*-mercaptoethanol (prohibits differentiation and is left out of the medium from this stage) for 2 days. 18 *μ*M cytosine arabinoside (Ara-C) was added on day 10 and the dishes were incubated for a further 2 days to optimize neuron-like cell numbers [40]. Immunocytochemistry was carried out on differentiated cells (day 12 of the assay) with NF200, a primary antibody specific to neurofilaments.

#### 2.6.2. Adipocyte Differentiation Assay

This was carried out according to established methods [41] and the resulting cultures were stained with oil red O to detect lipid [5].

#### 2.6.3. Endoderm Differentiation Assay

6 days after their formation, EBs were plated onto 35 mm 0.1% gelatin-coated dishes and maintained in ESC differentiation medium. After 12 more days, with medium changes every second day, the cells were fixed and stained with antibodies against albumin and alpha-1-fetoprotein.

### 2.7. Human ESC and iPSC Culture

The human H9 ESC line and iPSC established and validated in our laboratory were used in this study. Cells were grown on mitomycin-inactivated MEFs with hESC medium containing KnockOut DMEM, 100 *μ*M *β*-mercaptoethanol, 1 mM L-glutamine, 1% nonessential amino acids, 20% serum replacement (Invitrogen), 1% penicillin-streptomycin, and 8 ng/ml FGF2 (Invitrogen), which was changed daily.

### 2.8. Culture of hESCs and iPSCs on Dermal Feeder Layers from Human Skin

Initially, mitomycin-inactivated MEF or human DP, DS, and SF were seeded at 4 × 10^5^ cells per well of 6-well culture plates in dermal medium. After 1 day, hESCs or iPSCs were seeded onto the feeders and fed every day with hESC medium. hESC and iPSC cells were passaged every 4-5 days by incubation in 1 mg/ml collagenase IV (Invitrogen) at 37°C or mechanically dissociated and then removed to freshly prepared feeders. Cultures were fixed and incubated with nitro blue tetrazolium and 5-bromo-4-chloro-3′-indolyphosphate (NBT/BCIP) substrate solution to detect alkaline phosphatase activity. For immunocytochemistry analysis, colonies were separated from their feeders and seeded onto chamber slides coated with ESC-qualified matrigel in MEF-conditioned hESC medium for 2 days prior to fixation and analysis.

### 2.9. Flow Cytometry of hESCs

For flow cytometry analysis, hESCs were collected using collagenase IV treatment (1 mg/ml for 5 minutes) followed by brief accutase incubation. Cells were suspended in staining buffer (PBS + 5% FCS) at 10^6^ cells/ml. 10^5^ cells were stained with TRA-1-60, SSEA-4 (Millipore), or Oct4 (Santa Cruz) antibodies at 10 *μ*g/ml final concentration. Several washes were carried out in staining buffer before proceeding to staining with secondary antibodies. Cells were washed three times and resuspended in staining buffer before being analyzed with FACS Calibur (BD) using CellQuest. 10,000 events were acquired for each sample, and propidium iodide staining (1 *μ*g/ml) was used to distinguish live from dead cells.

### 2.10. Reverse Transcription Polymerase Chain Reaction

Total RNA from 2D and 3D cocultures, rodent DP and DS cell cultures, mESC cultures with or without LIF, and dissected vibrissa follicles (end bulb, mid-follicle, and upper follicle) were prepared using the ToTALLY RNA Kit (Ambion) as described by the manufacturer.

Contaminating genomic DNA was eliminated by DNase I digestion (DNA-free kit, Ambion). Approximately 1 *μ*g total RNA from each sample was reverse transcribed (Superscript II RT, Invitrogen) using oligo-dT primers. PCR was then performed using Taq polymerase (Invitrogen) with specific primer sets for each gene (Table 1). PCR reactions were carried out as follows: 94°C for 5 min; 20–30 cycles of 94°C for 30 s, gene-specific annealing temperature for 30 s, and 72°C for 60s; and 72°C for 5 min.

### 2.11. Immunocytochemistry

Cultured cells were fixed in either methanol (2 min, −20°C) or 4% paraformaldehyde in PBS (10 min, room temperature), blocked against nonspecific binding, and permeabilized with 0.1% triton X prior to incubating with primary antibodies (Table 2) for 1 hr at room temperature or overnight at 4°C. Following primary antibody incubation, cells were rinsed in PBS and incubated with secondary antibodies for 1 hr at room temperature in the dark. Stained samples were mounted under glass coverslips in Mowiol (Calbiochem) and visualized with a Zeiss Axiovert 135 microscope. Isolated rat vibrissa follicles were frozen in OCT (Agar Scientific) in liquid nitrogen or embedded in paraffin wax after overnight 4°C paraformaldehyde fixation. Antibodies bound on paraffin-embedded tissues were visualized using the VECTASTAIN ABC-AP kit (goat IgG, Vector Laboratories).

### 2.12. Bone Marrow Stromal Cell Culture

Mouse bone marrow stromal cell (BMSC) primary cultures were established as previously described [42]. The S17 stromal cell line is an immortalized cell line originally isolated from Dexter culture [43]. Cultures were routinely passaged as previously described and were plated into 35 mm dishes at p3 or p4 and allowed to grow to confluence.

### 2.13. Isolation and Purification of Haematopoietic Progenitors

Mice (6-week-old Balb/c) were killed by cervical dislocation, and bone marrow was collected from their femurs by flushing with PBS using a 25 g needle and 1 ml syringe. Red blood cells were lysed by incubation for 10 minutes in a hypotonic solution of ammonium chloride (7.5%) at room temperature, and the remaining nucleated cells were collected by centrifugation at 2000 rpm and washed 3 times in PBS. Viability was determined by Trypan blue exclusion, and cells were counted and resuspended in PBS. These were separated into c-kit-enriched or depleted populations by the magnetic activated cell sorter (MACS) system (Miltenyi Biotec, Bergisch-Gladbach, Germany).

### 2.14. Stromal Support Experiments

A summary of the strategy for these experiments is shown in the Supplementary Figure S1. Stromal support experiments used mouse DP and DS between passage 3 and passage 5 as stromal layers, with either primary mouse bone marrow stromal cells or S17 mouse stromal cell line as positive controls. All stromal cells were grown in MEM with 10% FCS. Confluent stromal layers were seeded with10^5^ unfractionated bone marrow nucleated cells. Alternatively, they were seeded with the c-kit-enriched (MACS-bound) population or with the depleted (MACS-run through) population from 10^5^ bone marrow nucleated cells. Cocultures were maintained for 28 days, and nonadherent cells were harvested every seven days and used for CFU-M assays. Experiments were repeated 6 times, cell numbers were entered into Excel, and standard deviations were calculated using Excel.

### 2.15. CFU-A Assays

CFU-A assays were performed essentially as previously described [44]. Briefly, support layers of 0.5 ml 0.6% agar in Alpha-MEM (Gibco-Invitrogen) containing 100 *μ*l per ml of conditioned medium from the cell lines AF1-19T (a source of GM-CSF) and L929 (a source of M-CSF) were plated into the wells of a 24-well plate. Cells (2000/well) were overlaid in 0.5 ml of plating medium (identical to support layer, but using 0.15% agar). Plates were cultured for 20 days in 10% CO_2_, in humidified incubators. Experiments were repeated 6 times, colony numbers were entered into Excel, and standard deviations were calculated using Excel.

## 3. Results

### 3.1. Expansion of mESCs Cocultured on Rodent DP or DS Cells

**Figure 1:**
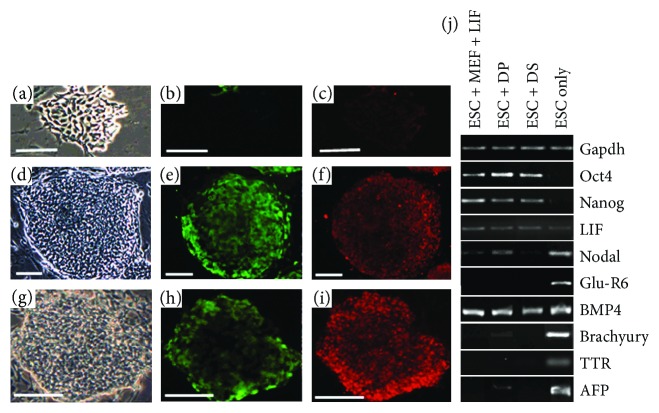
Maintenance of mESCs by rodent hair follicle dermal cells. Rex-1 EGFP-transfected CGR8 mESCs were cocultured with PVG fibroblasts (a–c), DP (d–f), and DS (g–i) cells. The ESCs lost colony morphology (a), Rex-1-directed EGFP expression (b), and Oct4 expression (c) when cultured with skin fibroblasts. However, mESCs were effectively maintained in an undifferentiated state by coculture with follicular dermal cells, both DP and DS, displaying compacted colonies (d, g), eGFP expression under the control of Rex-1 (e, h), and high levels of Oct4 expression (f, i). RT-PCR analysis of CGR8 mESCs cocultured with rat DP and DS cells (j). PCR products obtained using primers specific for mouse *GAPDH* (control), *Oct4*, *Nanog* (markers of undifferentiated ES cells), *LIF*, *BMP4* (involved in ES support), *Nodal*, *Glu-R6*, *Brachyury*, *TTR*, and *AFP* (markers of differentiation). Scale bars = 50 *μ*m.

When mESCs were cocultured with follicular dermal cells (Z40 or PVG in origin) in dermal cell medium, we observed growth of colonies of undifferentiated mESCs similar to those formed on MEFs when supplemented with LIF. The mESCs settled in discrete colonies on top of the dermal cell monolayer (Figures 1(a), 1(d), and 1(g)) and expressed EGFP under the control of Rex-1 (undifferentiated mESC marker, Figures 1(b), 1(e), and 1(h)). The rodent feeder cells were not mitotically arrested and were either split together with the mESCs every 2 or 3 days or maintained as a confluent layer under the mESC colonies. In multiple experiments, the longest continuous coculture of mESCs with rodent dermal cells was either 8 passages or 4 weeks (depending on the method used) before the cocultures were used for other tests. When mESCs were grown with rodent SF cultures, colonies became flatter and less distinct in appearance (Figure 1(a)), and lost Rex-1-directed GFP expression (Figure 1(b)) and Oct4 expression (Figure 1(c)). However, irrespective of the coculture methodology, mES cells grown on follicular dermal cells (DP or DS) showed no significant loss of colony morphology (Figures 1(d) and 1(g)), or Rex-1-directed GFP expression (Figures 1(e) and 1(h)), or evidence of differentiation as indicated by the pluripotency marker Oct4 (Figures 1(f) and 1(i)). After eight or more passages on rodent DP and DS cells, the expanded mES cells were separated from their cocultures and analyzed using RT-PCR for the gene expression of various ES pluripotency and differentiation markers. High levels of *Oct4* and *Nanog* expression were detected in mESCs when cultured on DP and DS cells, but not when cultured without LIF or feeder layers (Figure 1(j)). mESCs cocultured with DP and DS cells in dermal medium (particularly with DS cells) did not express significant levels of differentiation markers such as *Nodal*, *GLU-R6*, *Brachyury*, *AFP*, and *TTR* at any point, whereas these were highly expressed in mESCs cultured in ES differentiation medium for 6–14 days.

### 3.2. The Supportive Role of Rodent DP and DS on mESCs Is Mediated via Soluble Factors

**Figure 2:**
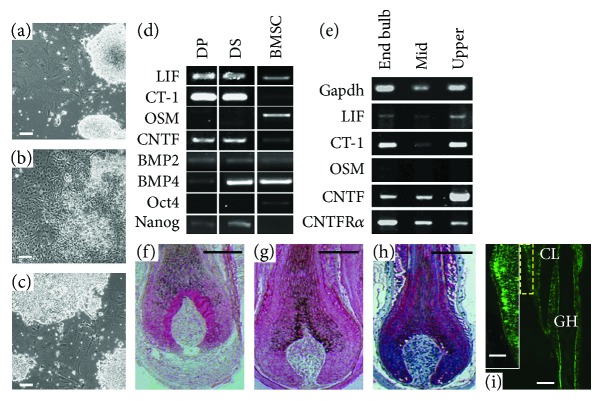
Dermal cells provide support for mESCs via soluble factors. mESCs were cultured in complete mESC medium (a), ESC differentiation medium (b), and DP/mESC coculture conditioned medium (c) for 10 days. Cultures maintained in conditioned medium showed maintenance of typical mESC colony morphology and behaviour. (d) Cytokines known to be involved in mESC maintenance are expressed by follicular DP, DS, and BMSC. (e) Several of these cytokines are also expressed in different regions of vibrissa follicles as detected by PCR. However, the localization of LIF (f), CNTF (g), and gp130 (h) by immunohistochemistry (pink staining) in the lower follicle bulb region shows that they are predominantly in the follicle epithelium. (i) gp130 is also expressed in the upper follicle (i) specifically in the bulge epithelium (i^∗^) (region delineated by a yellow rectangle) (CL: club hair; GH: growing hair). Scale bars (a–i) = 100 *μ*m and (i^∗^) = 50 *μ*m.

Having established that pluripotent mESCs could be maintained in an undifferentiated state by follicular dermal cells in coculture, we next investigated if this process was mediated by soluble factors. As previously observed, there are marked contrasts between mESCs maintained in complete mESC medium (dense colonies with just a few differentiated cells) and those cultured in ESC differentiation medium (numerous morphological changes) (Figures 2(a) and 2(b)). When we maintained mESCs in conditioned dermal medium from DP/ES cocultures, we found that this was most effective at maintaining undifferentiated mESC morphology, with DS/ES-, DP only-, and DS only-conditioned media being progressively less effective. However, in all conditioned medium cultures, a larger proportion of the mESCs maintained a typical mESC morphology than in the culture maintained in ESC differentiation medium (Figure 2(c)), suggesting that soluble factors secreted by the dermal cells (both in the presence and absence of ESCs) prevent differentiation of ESCs.

### 3.3. Expression of ES Cell Supporting Cytokines *In Vitro* and *In Vivo*

In an attempt to determine which soluble factors may be involved in mediating mESC maintenance, RT-PCR was used to detect transcripts of potential candidates known to be involved in mESC support; *Nanog*, *Oct4*, *LIF*, *CNTF*, *OSM*, and *CT-1* expression was examined in DP, DS, and BMSC which has previously been shown to support the expansion of undifferentiated ESCs [13]. Members of the bone morphogenetic family (BMP) have also been implicated in mESC regulation in combination with LIF [45]. *BMP2* was detected in DP and DS cells, while *BMP4* expression was typically lower in DP cells when compared with DS and BMSC (Figure 2(d)). Cultured rodent DP and DS cells expressed higher levels of *LIF*, *CT-1*, *Nanog*, and *CNTF* than BMSC, while Oct4 (at low levels) and *OSM* expression was seen in BMSC, but not in dermal cells. Conversely, *Nanog* was expressed in DP and DS cells but was not detectable in BMSC (Figure 2(d)).

To examine the *in vivo* expression of cytokines of interest, mid-anagen vibrissa follicles dissected from three Zin40 mice were used as a source of RNA. *CNTF*, *LIF*, *CT-1*, and the receptor component *CNTFRα* were expressed in all regions of mid-anagen follicles, while *OSM* was not expressed at comparable levels (Figure 2(e)). We investigated the localization of a subset of these cytokines in intact follicles by immunohistochemistry, and interestingly, we found that localization of LIF (Figure 2(f)) and CNTF (Figure 2(g)) was not only confined to dermal cell populations. Indeed, rather than being highly expressed in the DP and DS, LIF appeared to be concentrated in the epithelium immediately surrounding the DP, whereas CNTF appeared to have a more general distribution, with localized expression in the upper third of the DP and lower DS, coupled with higher levels of epithelial localization. Since the IL-6 family cytokines investigated here all act through a receptor complex containing gp130, we also examined gp130 distribution in intact follicles (Figure 2(h)). Again, this was present predominantly in the epithelial layers of the follicle, with distinct expression at the DP/epithelial boundary. Specifically in the bulge region higher up the follicle (Figure 2(i)), the epithelial cells showed strong gp130 immunolabelling (Figure 2(i^∗^)).

### 3.4. Follicular Dermal Cells Are Unable to Prevent ES Cell Differentiation in 3-Dimensional Cultures

**Figure 3:**
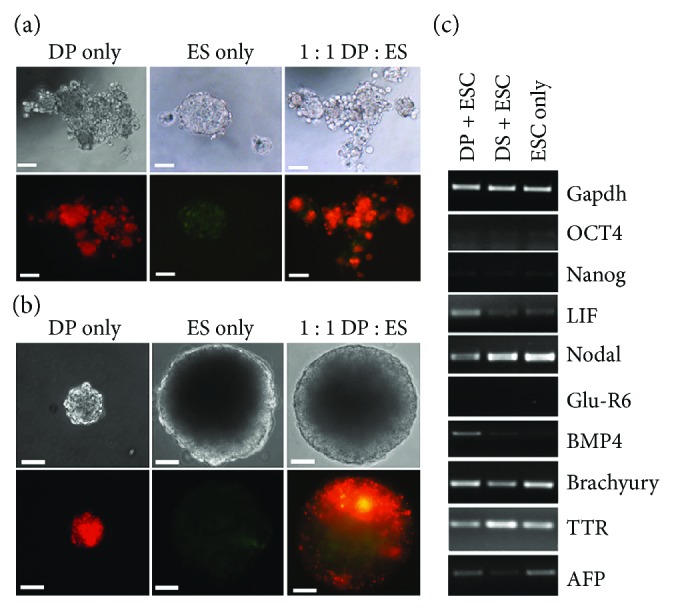
Coculture of dermal cells and mESCs in embryoid bodies does not prevent differentiation. (a) Embryoid-like bodies formed after 3 days in hanging drop culture. Follicular dermal cells were stained with DiI and mESCs expressing GFP under the control of the CAGG promoter. Hanging drops containing follicular dermal cells produced EBs having several foci of aggregation, in contrast to those containing ES cells alone. (b) EBs after 9 days in suspension culture. EBs containing mESCs were much larger than those with only follicular dermal cells. (c) Differentiation markers expressed by mESCs in 3 dimensional cocultures with follicular dermal cells. Scale bars (a) = 50 *μ*m and (b) = 100 *μ*m.

Having demonstrated that follicular dermal cells are able to inhibit mESC differentiation in 2D cultures, we next investigated if they can prevent differentiation of mESCs in 3D cultures. EBs were produced containing either mESCs only or a 1 : 1, 3 : 1, or 10 : 1 ratio of follicular dermal to mESCs by hanging drop culture. During the early stages of EB culture (2–4 days), it was noted that the inclusion of follicular dermal cells tended to modulate EB formation, with EB's containing DP or DS cells being less regularly shaped than those comprising mESCs alone, and often, with more than one EB forming in each hanging drop (Figure 3(a)). Once EBs were transferred to suspension culture, they continued to grow for 9–12 days, often producing large cysts containing cardiomyocyte-like (beating) cells. We found that this remained the case no matter how many dermal cells were included, although a higher proportion of dermal cells generally resulted in smaller cysts. Follicular dermal cells alone were unable to form cysts and instead were maintained as tight clumps of cells with little or no increase in size. Within EBs, it appeared that dermal cells were not well distributed and instead were remaining aggregated (Figure 3(b)). Surprisingly, however, in all cases, the mESCs rapidly outgrew the dermal cells and expressed differentiation markers not present in 2D cocultures (Figure 3(c)). We found that neither the addition of LIF nor the addition of an increasing proportion of dermal cells to the EBs could prevent this (data not shown); so, for all subsequent experiments, we used a 1 : 1 ratio of dermal : ESCs for EB formation. The mESCs rapidly lost expression of *Oct4* and *Nanog*, further confirming that undifferentiated mESCs were not maintained, although LIF expression was not substantially downregulated. We found that the pattern of differentiation markers expressed varied from that shown by differentiated mESCs in 2D culture (compare Figures 1(j) and 3(c), ESC only), with no evidence of *GluR6* expression and significant loss of *BMP4* expression by mESCs in the 3D cultures.

### 3.5. Cocultured mESCs Maintain Differentiation Potential in Culture

**Figure 4:**
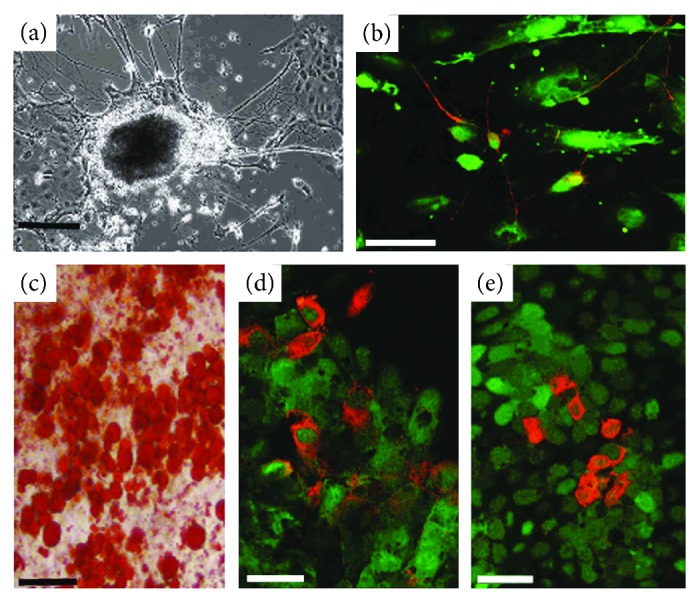
mESCs maintain pluripotency after coculture with dermal cells. (a) Networks of neurons extend from EBs derived from mESCs after 6-day coculture with PVG rat DS cells. (b) GFP mESCs after 6-day coculture with PVG DP cells produce neuron-like cells; the projections of which are visible by fluorescence microscopy. Immunostaining with anti-NF200 antibodies demonstrates expression of neurofilament (red) and the colocalization with the GFP fluorescence (green). (c) ESCs cocultured with PVG DS for 6 days produced high levels of lipid (red). One EB in particular was observed to be producing copious amounts of lipid. Endodermal cells are present in differentiated cells from 6-day cocultures of GFP mESCs with PVG DP (d) and Z40 DP (e). Green fluorescence identifies the cells as being of mESC origin while red fluorescence shows immunoreactivity for albumin (d) and AFP (e). Scale bars (a) = 200 *μ*m, (b–e) = 50 *μ*m, and (d) = 25 *μ*m.

When mESCs were directly differentiated in the neuronal differentiation assay, we observed networks of neuron-like cells after 12 days in culture. Axon-like projections extended in networks from EBs, which in association with other cell types adhered to the culture substrate (Figure 4(a)). Cells originating from cocultures with PVG and Z40, DP, and DS cells all gave positive results in this assay, with a minimum of 65% of EBs producing networks of neuron-like cells.

The greatest numbers of neuronal outgrowths were observed in cells originating from 6 day Z40 DS : mESC cocultures where 90% of EBs had neuron-like cells associated with them. Immunocytochemistry for neurofilament markers confirmed these cells to be neuronal, and colocalization with GFP also confirmed that they were derived from mESCs (Figure 4(b)).

In the adipocyte differentiation assay, mESCs isolated from PVG and Z40, DP, and DS cocultures gave consistently positive results. Throughout experimental cultures, oil red O staining showed small lipid droplets and large lipid-filled cells in patches ranging from a few cells to up to 50 cells closely packed together (Figure 4(c)). Approximately 30% of each culture exhibited high levels of lipid deposition.

In the endoderm differentiation assay, mESCs from all cocultures gave positive results. Small clusters of GFP-positive CGR8-GFP cells were immunoreactive for albumin (Figure 4(d)) and alpha-feta-1-protein (Figure 4(e)). As expected, these comprised no more than 1% of the total cell population.

### 3.6. Maintenance of hESCs and iPSCs by Hair Follicle Dermal Feeder Layers

**Figure 5:**
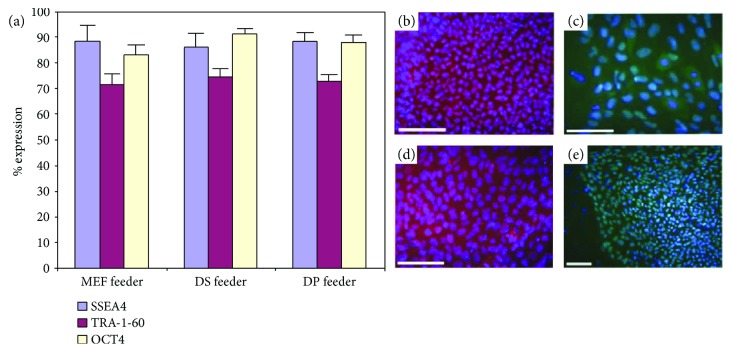
Follicular dermal cells support hESCs. hESCs cocultured with either MEFs, human DP cells, or human DS cells showed comparable levels of expression of SSEA-4, TRA-1-60, and Oct4 (a), as determined by flow cytometry indicating that the DP and DS cells were as capable of maintaining hESCs in an undifferentiated state as MEFs. Although all DP and DS cell lines tested were able to maintain hESCs expressing high levels of these markers, it was evident that some lines were more effective than others. hESCs maintained by MEFs and labelled for NANOG (b) and OCT4 (c) have similar staining to hESCs maintained by DP cells and labelled for NANOG (d) and OCT4 (e). Scale bars = 100 *μ*m.

**Figure 6:**
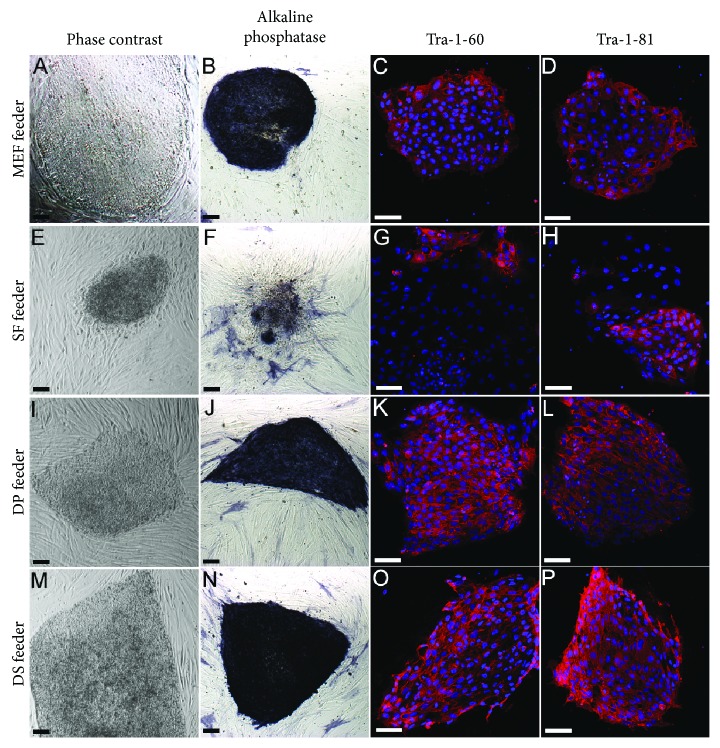
Hair follicle dermal cells maintain human iPSCs in an undifferentiated state. Human iPSCs were grown on either MEF, human SF, DP, or DS cells. On MEFs, iPSCs maintained normal ESC-like morphology (a), high alkaline phosphatase (b), Tra-1-60 (c), and Tra-1-81 (d) expression. In comparison, when grown on fibroblast feeders, the iPSC colonies became less compact (e), and with increasing passage number lost expression of pluripotency markers and alkaline phosphatase (f–h). When grown on hair follicle dermal cells, DP (i–l), but particularly DS (m–p), the iPSCs maintained normal ESC morphology and high levels of expression of ESC pluripotency markers for several passages in culture. Scale bars = 100 *μ*m.

After demonstrating that mESCs were maintained in an undifferentiated state by coculture with follicular dermal cells, we next asked if human hair follicle dermal cells could act as effective feeders for hESCs and iPSCs. When we cultured hESCs on mitotically inactive human DP or DS feeders for multiple passages, high levels of SSEA-4, TRA-1-60, and OCT4 expression were maintained, as determined by flow cytometry (Figure 5(a)). Although all DP and DS lines tested were able to maintain hESCs expressing high levels of these markers, it was evident that some lines were more effective than others. Immunolabelling of DP-supported hESCs with antibodies to NANOG and OCT4 showed levels of staining equivalent to those shown by hESCs cultured with MEFs (Figures 5(b)–5(e)). Subsequently, when we cultured human iPSCs on mitotically inactivated MEFs (Figures 6(a)–6(d)), human SF (Figures 6(e)–6(h)), DP (Figures 6(i)–6(l)), or DS (Figures 6(m)–6(p)), we found that they maintained a typical hESC-like appearance on the follicular dermal feeders, in discrete colonies with a high nuclear to cytoplasmic ratio. iPSC grown on MEFs, DP, or DS feeders for several passages remained positive for the pluripotency markers alkaline phosphatase, Tra-1-60, and Tra-1-81. Comparatively, expression of these markers was lost in iPSCs after a short period of growth on interfollicular SF feeders.

### 3.7. Hair Follicle Dermal Cells Support Haematopoietic Progenitors

All stromal cell types appeared to support haematopoietic cells, as indicated by the typical cobblestone morphology of the cells on the surface of the cultures (Supplementary Figure S2).

**Figure 7:**
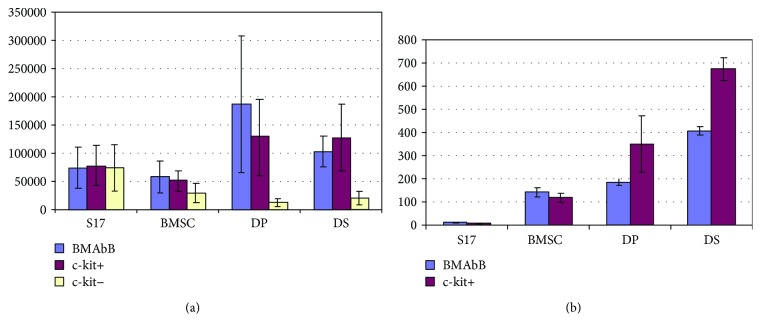
Hair follicle dermal cells support haematopoietic activity in coculture with murine bone marrow cells. (a) Total numbers of nonadherent cells produced by coculture of murine bone marrow cells with different stromal support cells over a 28-day period. *N* = 6 ± SD. (b) CFU-A colony production by coculture of murine bone marrow cells with stromal cells. Total burst colonies produced per sample over a 28-day period. No colonies were produced from c-kit fractions for any stromal condition. *N* = 6 ± SD. S-17: an immortalized stromal cell line; BMSC: bone marrow stromal cells; DP: dermal papilla cells; DS: dermal sheath cells.

Cell counts revealed that DP and DS cultures were at least as effective as bone marrow stroma and S17 cell lines in supporting the proliferation of nonadherent cells in coculture experiments (Figure 7(a)). This was particularly the case for unfractionated or c-kit-enriched populations. Flow cytometry with CD45 confirmed that haematopoietic cells were being produced (data not shown), but this could have been due to mitosis of mature cell types. In order to investigate whether haematopoietic progenitors were being supported by the stromal cultures, the nonadherent cells were subjected to colony assay using CFU-A.  Figure 7(b) shows that the number of CFU-A colonies produced following coculture with DP or DS cultures appeared greater than that from conventional Dexter-type bone marrow stromal cell culture. This increase in haematopoietic progenitor number was especially marked for the c-kit-enriched population.

## 4. Discussion

The initial hypothesis for the current work was that follicular dermal cells, when cocultured with pluripotent ESCs, might induce differentiation along the lineage of follicular epithelial cells. The inductive capacity of hair follicle dermal cells has been well documented [1, 2]. Nonfollicular epithelium will form follicles when associated with DP cells or embryonic dermis from hairy skin [3, 4], demonstrating the ability of DP cells to direct the differentiation of cells in close proximity, as is believed to be their physiological role in the adult hair follicle [1, 46]. In our hands, rat vibrissa follicle dermal papilla cells lose this inductive capacity around passage 4 in culture [47]. It has been shown that ESCs differentiate along both dermal and epidermal lineages to produce a tissue equivalent to embryonic skin when exposed to factors produced by skin fibroblasts [48]; so, it seemed reasonable to postulate that follicular dermis would induce differentiation along follicular lineages. In contrast to the working hypothesis, follicular dermal cells maintained both rodent and human ESCs and iPSCs in an undifferentiated state after long-term coculture. As both pre- and postinductive rat dermal papilla cultures showed the same influence on ES cells, it appeared that this phenomenon was not linked to loss of DP-inductive properties. The colonies of mESCs produced, either in mixed cocultures or in cultures where the dermal and ESCs were not in contact, were identical to those maintained by LIF or an MEF feeder layer (as confirmed by TEM (data not shown), immunocytochemistry, RT-PCR, and the use of Rex 1-EGFP CGR8 cells). Both human and mouse ESCs retained high levels of intrinsic Oct4 and Nanog expression, which are known to maintain pluripotency both *in vivo* and *in vitro* [49–51]. Human iPSCs cultured on follicular dermal cell feeders also retained high levels of the cell surface antigens Tra-1-60 and Tra-1-81, comparable to the levels expressed by iPSCs grown on MEF's, demonstrating that undifferentiated iPSCs can be effectively maintained by follicular dermal cells (both DP and DS). These markers of undifferentiated hESCs disappear rapidly upon differentiation [52], as was seen when the cells were grown on control fibroblast feeder layers. We also showed that mESCs retained their pluripotency after coculture by performing differentiation assays to induce differentiation into cell lineages derived from each of the three germ layers. The observation that in 3D coculture, follicle dermal cells were unable to prevent ES cell differentiation typical of embryoid bodies reflects the powerful influence of the 3D environment on ES behaviour and the fact that there was segregation of the two cell types within the structures. It may also be that in 3D, the dermal cells had a different secretory profile.

The behaviour of mESCs exposed to dermal cell- or coculture-conditioned media in 2D, or in cultures where the cells were physically separated by a 0.45 *μ*M filter, indicated that soluble factors were present in the media secreted by the dermal cells (both in the presence and in the absence of ESCs), with the capacity to inhibit differentiation. A similar study identified secreted factors in MEF-conditioned medium that could maintain undifferentiated hESCs in the absence of feeder cells [53]. Four members of the IL-6 family of cytokines (LIF, CNTF, CT-1, and OSM) have been shown to maintain undifferentiated mESCs *in vitro* via the LIFR/gp130/STAT pathway [24, 54–58]. Therefore, we interrogated the mRNAs of cultures and found that transcripts of three of these cytokines, *LIF*, *CNTF*, and *CT-1* were detected in dermal cells. Additionally, BMPs are known to cooperate with LIF to maintain mESC pluripotency [45], and BMP2 and BMP4 were detected in the hair follicle dermal cell culture. We also found that cultured DP and DS expressed *Nanog*, a downstream effector of the LIF/STAT3 pathway in maintaining mESC pluripotency [49]. Further, undifferentiated hESCs can be maintained without feeder cells by the presence of high levels of Nanog [51]. The expression of these cytokines *in vitro* raises the intriguing possibility that they may play a functional role *in vivo*. The localization of LIF and CNTF in the follicle end bulb suggests that they are physiologically relevant. Past studies have cited them as promoters and inhibitors of both differentiation and proliferation [27, 24, 54–57, 59–61], processes that occur predominantly in the follicle end bulb and are key to the cyclic nature of hair follicle activity. Moreover, we noted strong gp130 expression in the bulge region of the follicle outer root sheath, which houses the main epithelial stem cell population of the hair follicle. However, the relative lack of cytokine expression in the dermal papilla cells of the follicle bulb, but the presence of *LIF*, *CNTF*, and *CT-1* mRNA in all segments of the mid-anagen follicle, indicates a widespread function within follicles, rather than a specific role in follicular epithelial stem cell maintenance. Similarly, the expression of *CNTFRα* mRNA throughout the follicle suggests that members of the IL-6 family are unlikely to be involved specifically in maintenance of stemness in the surrounding epithelial cells by the follicular dermis. It appears that the mechanisms by which the dermal cells support ES cell maintenance have no obvious parallels with regulation of epithelial stem cell activities in the follicle. The contribution of the above cytokines to various aspects of hair follicle biology remains to be fully defined although there are reported connections between the gp130/JAK/STAT pathway and follicle activities. Interleukin-6 itself has been linked with hair growth inhibition and follicle regression [62, 63]. Moreover, in mice, JAK-STAT3 signalling is needed for the initiation of spontaneous anagen [64]. Stat5 activation in the follicle DP has recently been shown to trigger follicle entry in the growing phase (anagen) [65], and one of us (AMC) has recently shown that pharmacologically inhibiting the JAK/STAT pathway induced anagen from resting (telogen) follicles [66].

The elimination of animal material during both the derivation and long-term culture of hESCs or iPSCs is an important goal prior to application of these cells for clinical therapy. Animal-derived feeders and serum risk the introduction and transfer of nonhuman pathogens to human cells and increase the the risk of graft rejection when cells are introduced into patients [67,68].

A wide range of feeder cell types, conditioned media, and feeder-free systems has been investigated [25, 34], including a xeno-free system for derivation of hESCs using human serum and a human foreskin fibroblast feeder layer [69]. Human hair follicle dermal cells could be similarly utilized for both hESC derivation and long-term maintenance. Moreover, iPSCs have recently been derived from mouse hair follicle DP cells using a single transcription factor [70], and we have derived iPSCs from human hair follicle DP cells [71]. A recent report has shown that human follicle dermal (mesenchymal) cells maintain hES cells in an undifferentiated condition [72]. Our work supports this finding and extends it to human iPS cells. Therefore, follicle dermal cells may parallel the properties of human and mouse adipose-derived cells that can be used both to establish iPSCs in a feeder-independent manner and as feeder cells for supporting different pluripotent stem cells [73].

Previous studies have shown strong parallels between hair follicle dermal cells and bone marrow cells [17]. Therefore, our findings here that hair follicle dermal cells support haematopoiesis are to some extent unsurprising. However, the dermal cells were apparently equal to and possibly superior to bone marrow cells as stromal supports. Since we previously demonstrated that cultured hair follicle dermal cells can produce colonies in CFU-A assays and restore haematopoiesis in irradiated mice [8], this raises the question as to what extent the follicle dermal cells were contributing to the haematopoietic pool. This was not explored here and would need future work. Notwithstanding this, adipose-derived MSCs have also been reported to support haematopoiesis better than bone marrow cells [74]. A feature of the adipose cells was their expression of CXCL12 (SDF-1) a key regulator of haematopoiesis, which is also strongly expressed by cultured hair follicle dermal cells (see Supplementary Figure S2). It would be surprising if hair follicle dermal cells were to be routinely adopted for support of pluripotent human cells given the direction of travel towards feeder-free methods [25–31] and the availability of alternative human candidate cell types [32–35]. However, follicle dermal cells do fulfill important criteria, including being easily accessible, readily propagated, and efficient at maintaining undifferentiated hESCs. Moreover, where there is a clinical role for support cells in transplantation, as in haematopoiesis [74, 75], follicle dermal cells have an advantage. These cells are in the process of being exploited for use in the production of new follicles in the treatment of alopecia and the creation of improved skin grafts. Therefore, work on the safe bioprocessing of these cells for clinical transplantation is already in train, making their eventual use in the context of stem cell support a more plausible proposition.

## 5. Conclusions

Here, we have demonstrated that coculture of hair follicle dermal cells with ESCs or iPSCs can support their long-term maintenance. We further show that the follicle cells support haematopoietic activity. This could have potential benefit in a clinical context, where the elimination of animal feeder layers is necessary prior to application of pluripotent cells for therapy and where the application of mesenchymal stem cell-like populations goes beyond their own direct therapeutic use, to include a role as support cells for other transplantable cell types.

## Figures and Tables

**Table 1:** Primer sets for reverse transcription polymerase chain reaction.

Name	Sequence	Annealing temp (°C)	Size (bp)
GAPDH	F: GCC AAA AGG GTC ATC ATC TC	61	379
	R: ACG GAT ACA TTG GGG GTA GG		
Oct4	F: CCC GGA AGA GAA AGC GAA CT	58	362
	R: GAC GGG AAC AGA GGG AAA GG		
Nanog	F: AGG GTC TGC TAC TGA GAT GCT CTG	56	363
	R: CAA CCA CTG GTT TTT CTG CCA CCG		
LIF	F: ATT GTG CCC TTA CTG CTG CT	61	583
	R: GCC TGG ACC ACC ACA CTT AT		
CT-1	F: GAG GAA TAC GTG CAG CAA CA	57	389
	R: AGC ACC TTG GCT GAG AAG AT		
OSM	F: CAC GGC TTC TAA GAA CAC TGC	59	547
	R: CGA TGG TAT CCC CAG AGA AA		
CNTF	F: CTT TCG CAG AGC AAT CAC CT	61	579
	R: CCC CAT AAT GGC TCT CAT GT		
BMP2	F: TCC ATC ACG AAG AAG CCG TG	58	465
	R: CCA AAA GTC ACT AGC AAT GGC		
BMP4	F: AGG GCC AGC ACG TCA GAA TC	57	430
	R: ACC TTG TCA TAC TCA TCC AGG		
CNTFR*α*	F: CTG TTT CCA CCG TGA CTC CT	59	802
	R: TGG GAC ACT GGT CAA GAA GA		
Nodal	F: GCC AGA CAG AAG CCA ACT GTG	61	324
	R: TCA GAG GCA CCC ACA CTC CTC		
GluR6	F: CTG CAG CAC AGA GAG GAA CCA	60	488
	R: ATA ACT TCC TCC ATG TGC CTC AC		
Brachyury	F: GCT GAG ACT TGT AAC AAC CG	55	266
	R: GCA AAG GAC TCT GAT TAA CTG C		
TTR	F: CCG TTC CAT GAA TTC GCG GAT	60	240
	R: TTC ACG GCA TCT TCC TGA GC		
AFP	F: TTG CCT CCA CGT GCT GCC AGC	61	341
	R: CGC CAG CTG CTC CTC TGT CAG		

**Table 2:** Antibodies used during immunochemical analysis.

Antibody	Manufacturer	Dilution used
Anti-albumin	Dako	1 : 100
Anti-alpha-1-fetoprotein	Dako	1 : 100
Anti-*α*-smooth muscle actin	Sigma	1 : 10
Goat anti-CNTF	R&D Systems	1 : 40
Anti-GFP	Abcam	1 : 100
Anti-gp130	Santa Cruz	1 : 30
Goat anti-LIF	R&D Systems	1 : 100
Anti-NF200	Sigma	1 : 100
Anti-OCT-3/4	R&D Systems	1 : 20
Anti-Tra-1-60	Millipore	1 : 200
Anti-Tra-1-81	Millipore	1 : 200
FITC anti-mouse	DAKO	1 : 80
TRITC anti-mouse	Jackson Immuno	1 : 100
Alexa Fluor 546 anti-mouse	Molecular Probes	1 : 500
FITC anti-rabbit	DAKO	1 : 100
Alexa Fluor 546 anti-rabbit	Molecular Probes	1 : 500
TRITC anti-rat	Jackson Immuno	1 : 100
Alexa Fluor 594 anti-rat	Molecular Probes	1 : 500

## Abbreviations

DP: Dermal papilla
DS: Dermal sheath
ESCs: Embryonic stem cells
mESCs: Mouse ESCs
iPSCs: Induced pluripotent stem cells
EBs: Embryoid bodies
CM: Conditioned medium
BMSC: Bone marrow stromal cell
MEF: Mouse embryonic fibroblasts.

## Supplementary Materials

Supplementary Materials Supplementary material consists of a schematic illustrating the method and processes used for haematopoietic support assays and RT-PCR showing similarities in marker expression between hair follicle dermal cells and bone marrow cells. In particular, both express stromal cell-derived factor 1 (SDF-1) which has an important role in haematopoiesis. Supplementary Figure S1: schematic showing the strategy used to compare the support of hair follicle dermal cells and bone marrow stromal cells for blood cells. Supplementary Figure S2: RT-PCR demonstrates that important markers of the haematopoietic environment including, thrombospondin-1, V-CAM 1, and SDF-1 are expressed in bone marrow stromal cells (BM), S-17 an immortalized stromal cell culture line, and hair follicle dermal papilla (DP) and dermal sheath (DS) cells (two strains shown).
