# Supplementary material for: Hair Follicle Dermal Cells Support Expansion of Murine and Human Embryonic and Induced Pluripotent Stem Cells and Promote Haematopoiesis in Mouse Cultures
Source: Stem Cells Int. 2018 Aug 2;2018:8631432. doi: 10.1155/2018/8631432 (PMC6098861; doi:10.1155/2018/8631432)
Supplement: Supplementary Materials — Supplementary material consists of a schematic illustrating the method and processes used for haematopoietic support assays and RT-PCR showing similarities in marker expression between hair follicle dermal cells and bone marrow cells. In particular, both express stromal cell-derived factor 1 (SDF-1) which has an important role in haematopoiesis. Supplementary Figure S1: schematic showing the strategy used to compare the support of hair follicle dermal cells and bone marrow stromal cells for blood cells. Supplementary Figure S2: RT-PCR demonstrates that important markers of the haematopoietic environment including, thrombospondin-1, V-CAM 1, and SDF-1 are expressed in bone marrow stromal cells (BM), S-17 an immortalized stromal cell culture line, and hair follicle dermal papilla (DP) and dermal sheath (DS) cells (two strains shown). [file 8631432.f1.pdf]

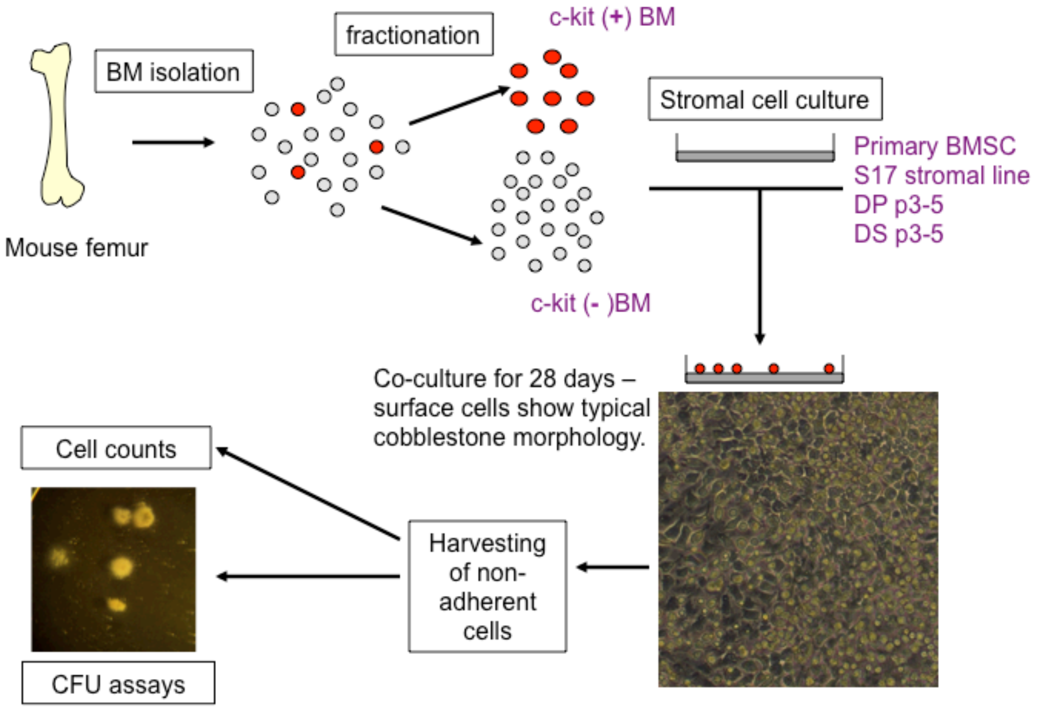

S1 – Schematic showing the strategy used to compare the support of hair follicle dermal cells and bone marrow stromal cells for blood cells.

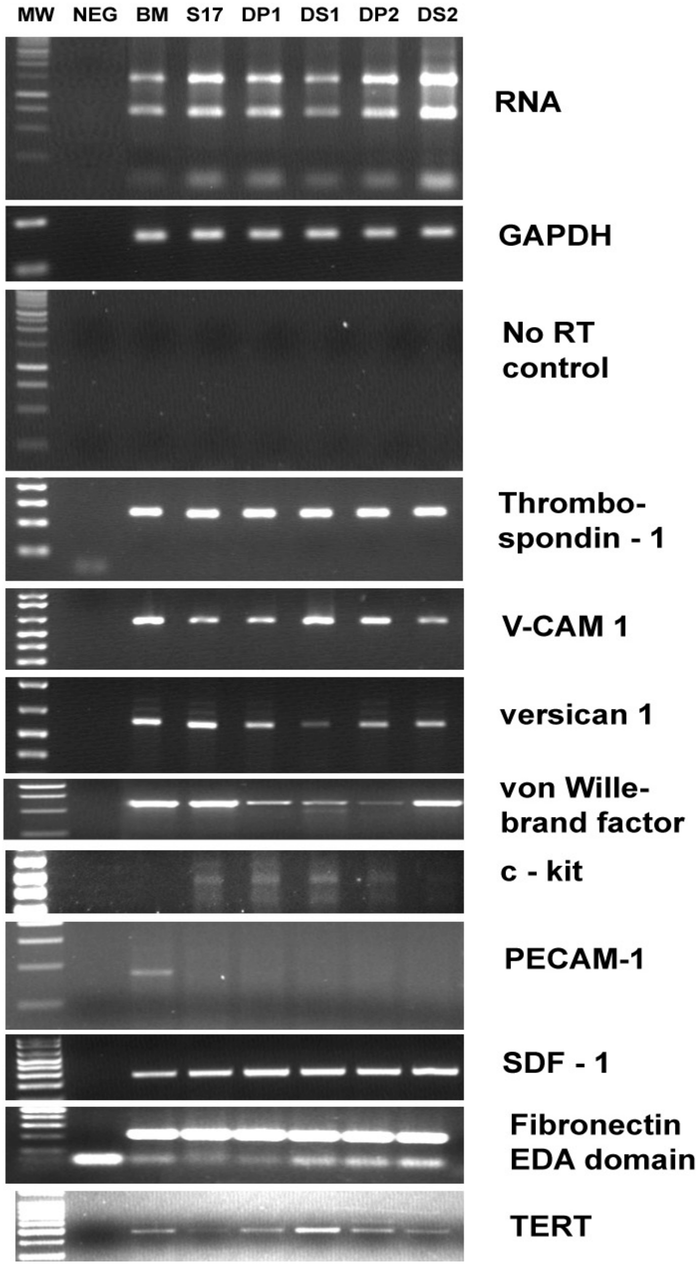

S2 RT-PCR demonstrates that important markers of the haematopoietic environment including, Thrombospondin-1, V-CAM 1 and SDF-1 are expressed in bone marrow stromal cells (BM), S-17 an immortalized stromal cell culture line, as well as hair follicle dermal papilla (DP) and dermal sheath (DS) cells (two strains shown).
